# Flexible Microfluidics: Fundamentals, Recent Developments, and Applications

**DOI:** 10.3390/mi10120830

**Published:** 2019-11-29

**Authors:** Hedieh Fallahi, Jun Zhang, Hoang-Phuong Phan, Nam-Trung Nguyen

**Affiliations:** Queensland Micro and Nanotechnology Centre, Griffith University, Brisbane, QLD 4111, Australia; hedieh.fallahi@griffithuni.edu.au (H.F.); jun.zhang@griffith.edu.au (J.Z.); h.phan@griffith.edu.au (H.-P.P.)

**Keywords:** flexible microfluidics, flexible microfluidic functions, microfluidic wearable electronics, flexible microfluidics in biology

## Abstract

Miniaturization has been the driving force of scientific and technological advances over recent decades. Recently, flexibility has gained significant interest, particularly in miniaturization approaches for biomedical devices, wearable sensing technologies, and drug delivery. Flexible microfluidics is an emerging area that impacts upon a range of research areas including chemistry, electronics, biology, and medicine. Various materials with flexibility and stretchability have been used in flexible microfluidics. Flexible microchannels allow for strong fluid-structure interactions. Thus, they behave in a different way from rigid microchannels with fluid passing through them. This unique behaviour introduces new characteristics that can be deployed in microfluidic applications and functions such as valving, pumping, mixing, and separation. To date, a specialised review of flexible microfluidics that considers both the fundamentals and applications is missing in the literature. This review aims to provide a comprehensive summary including: (i) Materials used for fabrication of flexible microfluidics, (ii) basics and roles of flexibility on microfluidic functions, (iii) applications of flexible microfluidics in wearable electronics and biology, and (iv) future perspectives of flexible microfluidics. The review provides researchers and engineers with an extensive and updated understanding of the principles and applications of flexible microfluidics.

## 1. Introduction

Microfluidics is the science and technology of handling small amounts of liquids [1,2]. Microfluidic devices typically consist of miniaturised components for fluid and flow manipulation such as microchannels [3], microvalves [4], micropumps [5,6,7], micromixers [8,9,10,11], and micro separators [12,13]. Microfluidics has found applications in a wide range of areas. For instance, in chemistry, employing microfluidics improves material synthesis, particularly nanomaterials and nanostructures because the interface between the reagents is larger relative to their volume [14,15,16,17]. In biology, microfluidics has been utilised for molecular analysis [18,19], cell analysis [20,21], and cell sorting [22]. In drug delivery, neural probes and implantable devices have integrated microfluidic components that can precisely deliver small amounts of medicine into specific targets within the body [23,24,25,26]. In electronics, microfluidics has been used as sensors, particularly for wearable systems [27,28,29,30]. Microfluidics has successfully proliferated in the above fields due to several reasons. First, microfluidics only requires a small amount of the sample liquid. This feature is vitally important in biology and medical applications, in some cases only a small quantity of sample material is available for analysis. Furthermore, only a trace of some rare particles may be available in specialised sampling across some unique areas, for example circulating tumour cells in the blood [31]. Microfluidics are of great help in this regard as they can sense and analyse even smallest amounts of liquids. Second, microfluidic devices could be integrated into a variety of systems, enabling an automated sample-to-answer analysis with less human intervention [32]. Third, microfluidic systems are often portable and can be used for point-of-care applications or deployed in remote locations [33,34].

A wide range of materials have been used for the fabrication of microfluidic devices. Historically, glass and silicon were the dominant choices due to their availability and advanced micromachining processes [35,36,37,38]. However, these materials were relatively expensive to process, brittle, and not self-sealing. The drawbacks associated with silicon and glass led to the emergence of new flexible materials with the ability to deform, bend, and stretch under mechanical loads for manufacturing the next generation of microfluidic devices. Depending on the application, microfluidic devices may require a unique set of properties from the material such as flexibility. Flexibility has been employed in many microfluidic components including microchannels, microvalves, micropumps, micromixers, and micro separators. Flexibility has many influences on microfluidic functions. For instance, flexibility leads to the change of the cross section of a microchannel when a fluid is passing through it [39]. This deformation, in turn, leads to changes in pressure distribution throughout the channel, affecting the flow pattern and associated microfluidic functions [40]. Additionally, the interactions between the flexible walls and the fluid lead to instabilities that result in new phenomena and application opportunities [41]. While flexibility has been widely employed in several device applications, the role of flexibility has not been systematically analysed. In other words, the role of flexibility upon the physics behind a microfluidic function has not been comprehensively discussed in the literature. Therefore, an in-depth understanding of the influence of flexibility on microfluidic functions is imperative. In this paper, flexible microfluidics refers to devices that are entirely made of a flexible material that can deform under an applied stress.

The convergence of flexibility and microfluidics has benefited many areas including biomedical devices and flexible wearable electronics. These two application areas require close contact with the human body and other biological systems. Flexible body-worn sensors based on microfluidics are attracting attention, since they have multiple advantages over other wearable sensors [42,43,44]. For making flexible electronics, conductive metal structures are usually deposited on or embedded in a flexible substrate. However, since the metal structure is solid, the sensor may face reduced sensitivity or even lose its function, after being exposed to repeated deformations. Microfluidics provides the opportunity of employing a conductive liquid inside a microchannel that will not lose functionality under multiple deformation conditions [44,45]. In life sciences, flexible microfluidics can mimic body organs and allow for in-depth in-vitro analysis of the cells [46,47,48,49,50]. That being said, flexible microfluidics is an emerging research field that needs to be introduced, studied, and further exploited. 

To date, no comprehensive review of flexible microfluidics exists in the literature. The existing reviews on microfluidics rarely focus on the aspects of flexibility in microfluidics [51,52,53]. This paper provides an extensive overview of the fundamentals and recent developments of flexible microfluidics as well as addressing the research gaps in this field. Here, we have only considered the references on microfluidic devices, where flexibility has a functional significance. Readers may refer to [54,55] for emerging research fields in microfluidics such as digital manufacturing and droplet-based microfluidics.

The present paper consists of four sections. The first section provides an overview of the materials for the fabrication of flexible microfluidics as well as their properties. For each material, examples of microfluidic devices are provided. A brief summary of the fabrication procedures and techniques have also been described. The second section discusses the fundamental physics of flexible microfluidics. This section covers the research gap regarding the effect of flexibility on microfluidic functionalities including channel deformation, mixing, separation, valving, and pumping. This section also provides examples of flexible microfluidic components for these fluid handling tasks. The third section summarises the recent applications of flexible microfluidics, specifically in wearable devices and life sciences. The last section discusses the perspectives of flexible microfluidics and points out the possible future research directions.

## 2. Materials for Flexible Microfluidics

### 2.1. Material Properties

**Figure 1:**
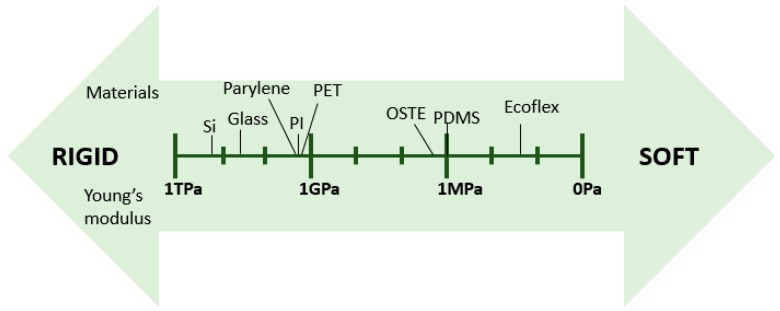
Young’s modulus spectrum of materials used for flexible microfluidics.

Recent applications of microfluidics require materials that are flexible. Flexible materials for microfluidics may need to be elastic as well, since they should return to the original shape after removing the stress. Many polymers can fulfil this need. Flexibility and elasticity of polymers are defined by Young’s (elastic) modulus and the shear modulus. Polymers exhibiting a lower elastic or shear modulus have a higher flexibility. Figure 1 lists the common materials used for flexible microfluidics fabrication and their corresponding Young’s moduli spectrum. Although flexibility and elasticity of a material is an intrinsic property, it could be adjusted to some extent by their preparation method. 

Polymers can be rigid, brittle, flexible, or elastic depending on their structures, synthesis method, and preparation techniques. The type of polymer and the fabrication method can be tailored according to a specific application. Polymers commonly used for microfluidic applications are classified as thermosets, thermoplastics, elastomers, and thermoplastic elastomers [51]. Thermosets are irreversibly crosslinked polymers that are rigid and stiff [56]. Thermoplastics could be brittle or flexible depending on the content of the amorphous and crystalline regions, their thickness, and their preparation techniques [57]. On the other hand, elastomers are intrinsically flexible and stretchable, and these properties could be adjusted by crosslinking content [58]. Elastomers also have the ability to reconfigure themselves to distribute the load and can go back to their initial state due to the covalent crosslinks among the long chains. Thermoplastic elastomers are copolymers consisting of both thermoplastics and rubbers and have the features of both groups [59].

Among these four types of polymers, the last three are of particular interest for flexible microfluidics. A flexible microfluidic device requires all layers to be constructed from flexible materials. As such, both the cover layer and the bottom layer with microchannels need to be flexible. There are two main approaches to fabricate a flexible microfluidic device. In the first approach, the channel-constructing and the channel-sealing layers are made of two different flexible polymers. The second approach uses a single material to form all layers of the device. The polymers used for flexible microfluidics are discussed as follow.

### 2.2. Siloxane Elastomers

Siloxane elastomers, also known as silicone rubbers or silicone, refer to synthetic polymers with a dimethylsiloxane repeating unit ((Si(CH_3_)_2_O)_n_) along their backbone. Poly(dimethylsiloxane) (PDMS) is the most widely used material for microfluidic fabrication and is the most recognised type of siloxane elastomers [60]. PDMS is an elastomer with long chains, and relatively low glass transition and melting temperatures, resulting in high flexibility and elasticity [61]. PDMS exhibits a low shear modulus of 250 kPa and thus behaves as a flexible rubber [62]. Flexibility of PDMS can be tuned by altering the ratio of the monomer to the crosslinker as well as controlling the thickness of the fabricated device. PDMS exhibits high optical transparency [63] making real-time observation possible. Optical access is extremely important not only for developing lab-on-a-chip devices but also for understanding physical and biological processes within these devices [64,65,66,67,68]. The above mentioned properties along with durability [69] and gas permeability [70] make PDMS an ideal material for flexible microfluidics. Furthermore, PDMS can be easily bonded to other substrates using plasma surface treatment [71]. Despite its great benefits for microfluidics, PDMS suffers from issues such as hydrophobic nature, being permeable to some liquids and gases, and incompatibility with several solvents [72]. The hydrophobic nature of PDMS, which is not favourable for applications that require surface adhesion such as cell culture [73,74], can be overcome by applying oxygen plasma to make the PDMS surface hydrophilic due to polar silanol groups, leading to better wetting [75,76]. Ecoflex rubber is just another commercial brand for siloxane elastomers and has been used in making microfluidic sensors to obtain more flexibility and stretchability [27,77,78]. 

**Figure 2:**
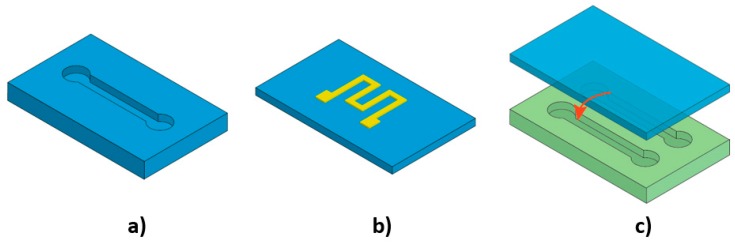
Schematic illustration of the three ways siloxane elastomers have been employed in flexible microfluidics: (**a**) A layer containing the microchannels; (**b**) Substrate for the electrodes; (**c**) Sealing layer.

Figure 2 illustrates the three different ways siloxane elastomers can be utilised in flexible microfluidics including a layer containing microchannels [46,79], a substrate for electrodes to be deposited on, and a sealing layer [46]. Yeo et al. reported the fabrication of a tactile sensor based on siloxane elastomers. In this work, the microchannels were constructed out of Ecoflex rubber and the electrode layer was out of PDMS. Interestingly, experimental results suggested a stronger bonding of Ecoflex–PDMS than that of Ecoflex–Ecoflex and PDMS–PDMS [77]. Furthermore, several flexible microfluidic devices have been fabricated entirely out of PDMS, including a flexible 3D microfluidic device with PDMS-based valves and pumps developed by Jeon et al. [80], a shear force sensor reported by Yin et al. [81], and capacitive pressure sensors created by Wong et al. [82]. The 3D printing technique has also been employed to form an entire PDMS-based microfluidic device [83]. 

### 2.3. Parylene

Parylene is a thermoplastic polymer that has been employed for the fabrication of flexible microfluidics [84]. Taking advantage of flexibility, biocompatibility, and low water absorption, parylene-based flexible microfluidic devices with biomedical applications such as injection tools, neural probes [85,86,87], implantable 3D electrodes for drug delivery [88], tunable microfluidic lens arrays [89], and inertial separators [90] have been produced. Flexibility of parylene can enable the implanted microdevices to follow the deformation of the tissue. Although parylene has a higher Young’s modulus compared to PDMS, it can be formed as an ultrathin film, thereby reducing the bending stiffness. For instance, in some flexible microfluidic devices, a thin perylene layer deposited on thin PDMS microchannels offers excellent flexibility while still rigid enough to retain the cross-sectional shape of the microchannels [90].

### 2.4. Poly(ethylene terephthalate) (PET)

PET is a thermoplastic polymer that has been used in microfluidics as a flexible platform. By adjusting geometrical properties such as the thickness and manipulating the moulding procedure of PET (controlling the amorphous and crystalline contents), highly flexible PET substrates could be achieved. PET has good gas and moisture barrier properties and is chemically inert. Yeo et al. fabricated a flexible, wearable microfluidic tactile sensor with high sensitivity. In this work, the microstructure was patterned on an Ecoflex layer, which was bonded to a flexible PET substrate with printed silver electrodes. The microchannels were subsequently filled with a conductive liquid alloy that functioned as the sensitive element [27]. Recently, Lin et al. [91] employed laser cutting to fabricate flexible and wearable 3D microfluidic devices using transparent PET films. The team developed a simple, low-cost, and scalable method for fabricating wearable microfluidics. Demuru et al. [92] reported a flexible microfluidic platform made of PET. Electrochemical sensors were integrated into a PET platform patterned via CO_2_ laser machining. The layers were later bonded using a silicone adhesive.

### 2.5. Poly(imide) (PI)

PI is another polymer that has been extensively used for flexible microfluidics. Its biocompatibility, good chemical properties, and high thermal stability make PI a good candidate for flexible microfluidic. Common fabrication processes for PI-based flexible microfluidic are lamination and sacrificial layer techniques [93,94,95]. In these processes, the microchannels were manufactured using dissolvable materials, which were later washed away. Such devices have applications in selective drug delivery to bio-tissues as well as lab-on-a-chip devices [96]. Additionally, fabrication of flexible porous microfluidic PI membranes has been reported for filtration and separation purposes [97]. 

Due to its biocompatibility, PI-based flexible microfluidic devices are suitable for medical applications, and have been used in biosensors and point-of-care (POC) systems [98]. Implantable microprobes have also been built using PI [99]. Zulfiqar et al. fabricated flexible microfluidic devices using partially cured PI with the microchannels formed by dry etching. A similar fabrication approach was also utilised in other POC devices as well as integrated microelectronics [100]. As PI has good sealing properties, flexible PI diaphragms have been used for micropumps [101,102]. Komatsuzaki et al. fabricated an all PI-based flexible micropump employing hot embossing [103]. Furthermore, chemical inertness makes PI a suitable material for the development of organic microreactors, where PDMS cannot be used due to low chemical stability. As such, by employing photoablation of PI films, flexible microreactors with good solvent resistance as well as excellent chemical durability have been manufactured by Min et al. [104].

### 2.6. Off-Stoichiometry Thiol-ene

Off-stoichiometry thiol-ene (OSTE)-based platforms have recently been used for the construction of flexible microfluidics [105,106,107]. This UV curable polymer comprises of excess unreacted thiol or allyl groups that can later be bonded together under UV exposure. As such, OSTE-based microfluidics can be formed through soft lithography. Chen et al. reported the construction of a flexible microfluidic device for electrochemical detection based on OSTE polymers [108]. Their proposed method for fabricating biosensors is cost-effective, pump-free, scalable, and could be mass-produced via roll-to-roll fabrication system using inexpensive polymers. 

### 2.7. Others

Various polymeric materials have been incorporated into siloxane elastomers either as a reinforcing agent or as another layer to overcome the drawbacks associated with silicones. These polymers include poly(tetrafluoroethylene) (PTFE), poly (vinylidene fluoride) (PVDF), parylene, PI, etc. PTFE has been used to reinforce PDMS flexible microfluidics when both flexibility and robustness are needed [48]. PVDF has piezoelectric properties and has been integrated into flexible microfluidic sensors [109]. Poly(3,4-ethylenedioxythiophene):poly(styrene sulfonic acid) (PEDOT:PSS) is a transparent conductive polymer that has also been incorporated into flexible microfluidic sensing platforms [110]. Thermoplastic polyurethane (TPU) is a low-cost, transparent, flexible material that has recently been used for microfluidics 3D printing through fused deposition modelling. Using TPU, microchannels as small as 50 μm were prototyped in a short time [111]. Furthermore, paper has also been utilised and studied for microfluidic fabrication [112,113]. However, paper material is only bendable but not stretchable; therefore, this review will not consider paper as a material for flexible microfluidics. Table 1 summarises materials used for fabrication of flexible microfluidics, their properties, advantages, and disadvantages.

## 3. Flexibility and Microfluidic Functions

**Figure 3:**
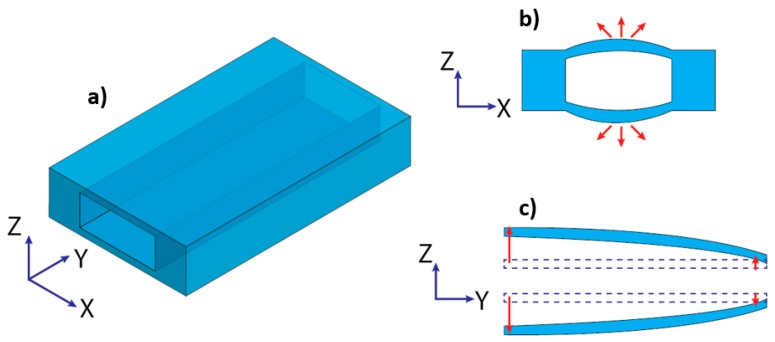
Schematic image of a flexible microchannel: (**a**) Channel before deformation; (**b**) Deformation of the channel cross-section perpendicular to the flow; (**c**) Deformation of the channel cross-section parallel to the flow.

Flexible and soft channel walls affect the physics of microfluidic components and also their functions. In the microscale, various aspects of flow physics such as rheology, flow resistance, surface tension, diffusion, and surface area become more significant [18]. On one hand, flexibility makes microchannels susceptible to deformation under high pressure or a high flow rate [120], resulting in a significant change in the size and geometry of microchannels. On the other hand, flow velocity, mixing, residence time, dispersion and separation are strongly affected by geometry and dimension variation of the microchannel induced by the channel deformation [40]. Figure 3 illustrates the typical deformation of a flexible microchannel under pressure-driven flow. Bulging of the channel walls alters the cross-sectional geometry. The deformation varies along the channel length and the maximum deformation occurs at the inlet of the microchannel, where the pressure is largest.

The flow pattern in flexible microchannels is influenced by the interactions between the fluid flow and the channel wall [121,122] as well as the hydraulic resistance. Hydraulic resistance is determined by the viscosity of the fluid, the geometry and the dimensions of the channel [123]. Flexible channels with deformable walls exhibit a nonlinear hydraulic resistance that is a function of the applied pressure or flow rate, in contrast to the flow-independent hydraulic resistance of a rigid channel. The following sections discuss the effect of flexibility on microfluidic parameters including flow rate and pressure drop as well as microfluidic functions such as mixing, valving, pumping, and separation. 

### 3.1. Effect of Flexibility on Flow Rate and Pressure Drop

Hardy et al. reported that the pressure drop in a deformable channel at a given flow rate is less than that in a rigid channel [39]. The pressure-drop in flexible microfluidics decreases because of the increase in the cross-sectional area of the deformable channel. Deformation of the channel is only possible with elastic materials. Fluid can pass through a deformable channel with a higher flow rate with minimum leaking because of the lower pressure build-up and the self-sealing characteristics as compared to a rigid channel. For a given pressure drop, a higher flow rate and consequently a higher throughput can be achieved with soft microchannels due to the decreasing hydraulic resistance with increasing cross-sectional area [124]. 

Effects of soft and flexible microfluidics on flow rate and pressure drop have been utilised for the development of a number of unique microfluidic devices, particularly to mimic biological systems such as blood vessels, lung, gut, and organs on a chip. Researchers have studied the behaviour of deformable microchannels under a pressure-driven flow and modelled the corresponding flow physics. Due to the complex interactions between the flow and the flexible walls, modelling the flow physics is a difficult task. As such, to simplify the problem, modelling of the microchannels typically views them as either a conventional type, with one rigid wall, or as a compliant type with all flexible walls. Adzima et al. observed the deformation of flexible microchannels under a pressure-driven flow [125] and developed a theoretical model to explain this phenomenon. 

Gervais et al. developed a simple model to predict the flow behaviour of a shallow soft microchannel with a wall thickness of more than 6 mm [123]. This model is valid for high aspect ratio channels. Later Cheung et al. improved this model for a wider range of aspect ratios [126]. Hardy et al. created a model for thin-walled flexible microchannels and studied the effect of wall thickness on equation parameters [39]. Raj et al. investigated the physics of fluid-structure interactions and derived a theoretical model for wall thicknesses below 100 micrometres [40]. Later, the team designed a robust model for the pressure distribution and deformation of the microchannel [127]. These models assume a parabolic deformation of the microchannels. However, this parabolic deformation does not fit all wall thicknesses and flow rates. Recently, Christov et al. presented a model without using the parabolic deformation. The authors considered fluid-structure interactions and employed perturbation theory with lubrication approximation [124]. Shidhore et al. provided a detailed report on modelling different types of elastic deformations such as bending and stretching and achieved a good alignment between the theoretical and experimental data [128].

### 3.2. Effect of Flexibility on Mixing

Due to the small size of microchannels, the Reynolds number (the ratio between inertial effect and viscous effect) is small, therefore making the flow laminar and stable in microchannels. Two fluid streams flowing side by side form an interface, where mixing occurs through molecular diffusion [129]. Thus, mixing in microchannels is a relatively slow process. To achieve efficient mixing in a short microchannel, passive and active mixing are the two basic strategies. Passive mixing is the scheme without external energy and relies on hydrodynamics to increase the contact area of the two fluids to be mixed. Passive micromixers with various channel configurations have been designed for promoting chaotic advection. Active mixing is the other scheme that requires external energy to induce local in situ mass transport for mixing enhancement. 

**Figure 4:**
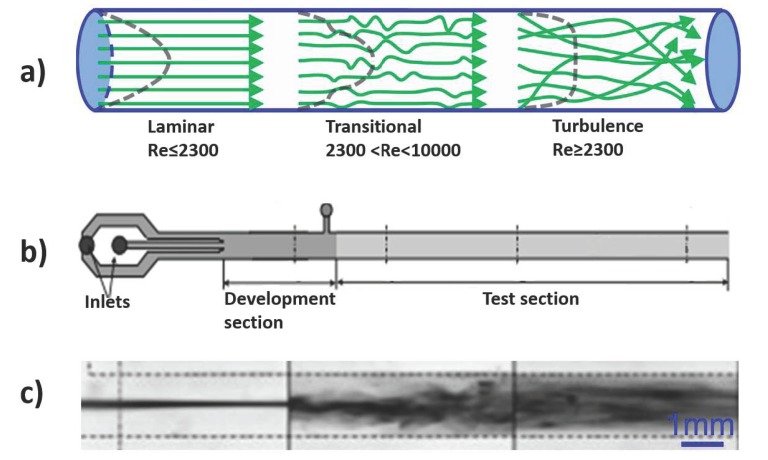
Mixing enhancement in a flexible microchannel caused by the instabilities induced by soft-wall fluid interactions: (**a**) Schematic presentation of the transition from laminar to turbulent flow; (**b**) Schematic of the flexible microfluidic mixing device; (**c**) Die-stream experiment of soft microchannels.

Flexible and soft walled microchannels have been used for mixing enhancement. Kumaran et al. extensively investigated the effect of soft-wall microchannels on mixing enhancement [130]. The interactions between the flexible channel wall and the fluid flow lead to flow instability and chaotic advection at a relatively low Reynolds number. In a rigid microchannel, the transition from laminar to turbulent regime takes place at a critical Reynolds number (the specific Reynolds number at which a transition from laminar to turbulent flow occurs). However, flexible microchannels allow for coupling between the soft wall and the fluid flow, leading to earlier instabilities and enhanced mixing at a much lower Reynolds number, as low as 200 [41,131,132,133]. Kumaran et al. defined a dimensionless parameter $\Sigma =\rho Gh^{2}/\eta^{2}$ that affects the critical Reynolds number, where ρ, *G*, *h*, and *η* are the fluid density, the elastic modulus of the wall material, the characteristic length, and the viscosity of the fluid, respectively. From the definition, it can be seen that the ratio of the elasticity of the wall material to the viscosity of the fluid affects the critical Reynolds number [134]. Figure 4 depicts the mixing experiments conducted in soft-walled microchannels [135] where flexible channels resulted in instabilities at a low critical Reynolds number leading to enhanced mixing [134].

In addition to the fluid-structure interaction, the cross-sectional area of the soft microchannel increases fractionally due to the significant deformation of the channels under pressure of the flow. Flexible microchannels offer two advantages for mixing enhancement. First, flexible microchannels allow for inducing flow instabilities, resulting in a low critical Reynolds number. Second, the notable increase in the cross-sectional area reduces the pressure drop. Both advantages promote a spontaneous transition to turbulence, resulting in enhanced mixing. Mixing using flexible microchannels has many applications in different areas such as multi-step reactions for nanoparticle synthesis. Vera and Kumaran recently investigated the effect of enhanced mixing on morphology and other characteristics of materials synthesized in soft-walled micromixers [136]. The authors reported that microfluidic mixing decreased the ripening time of the gold nanoparticles to a great degree. Flexible micromixing led to the synthesis of monodisperse particles, which is not possible in a conventional batch system. 

### 3.3. Effect of Flexibility on Valving

One of the key components for timing and controlling fluid flow in a microfluidic system is the microvalve [137]. Microvalves regulate the flow of fluid in the binary mode of either on and off or in a continuous mode. Many applications in life sciences and chemistry use microvalves simply as on/off switches, flow regulators and for sealing of liquids, gases, or vacuums [138]. Based on the operation state, microvalves can be classified as either normally closed or normally opened valves. According to the type of actuation, microvalves are classified into two main categories: Active and passive microvalves. Passive valves, also called check valves, use hydrodynamic pressure to rectify the flow in one direction. Active microvalves require external energy and are commonly controlled by magnetic, electric, piezoelectric, or thermal actuators [4]. While active microvalves require complex design and fabrication processes, passive valves are relatively simple in design and easy to be integrated into a microfluidic system. 

**Figure 5:**
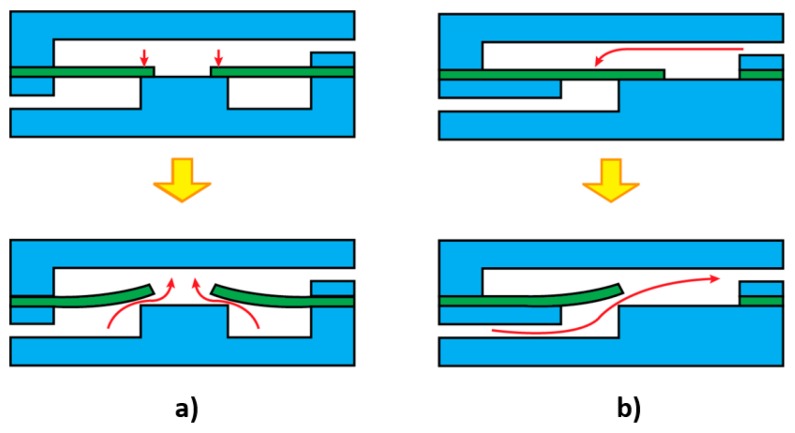
Schematic illustration of two types of flexible microvalves entirely made of poly(dimethylsiloxane) (PDMS): (**a**) Diaphragm valve; (**b**) Flap valve.

Preferred specifications for a microvalve include low power consumption, fast response, small dead volume, no leakage, and resistance to high-pressure. However, it is not always possible to fabricate a microvalve with all these desired features. Depending on the application, a compromise must be met among these characteristics. In the early stages of microfluidic technology, microvalves were mainly made of silicon. Later, soft materials were introduced for better sealing and ease of actuation. A typical example highlighting this is the elastomer-based microvalve, which was fabricated by a multilayer soft lithography process as reported by Quake et al. [139]. Since the elastomer has a low Young’s modulus, a large deflection could be achieved with a relatively small actuation power. Jeon et al. [80] presented a technique for the fabrication of two types of passive valves-flap valves and diaphragm valves-in microfluidic systems that are entirely made of PDMS as shown in Figure 5. Soft microvalves have many advantages over silicon-based valves such as smaller device footprint (valves are small relative to the microchannel), ease in fabrication, scalability, biocompatibility, cost-effectiveness, and most importantly no leakage (i.e., complete sealing) due to the conformality of the elastomeric layer. In addition, advanced microfluidic applications in biomedical and biochemical fields require an integrated all-elastomeric microfluidic device. Active valves limit the application range of microfluidic devices because they require external power. However, soft valves that are embedded in the device can be internally controlled, which benefits the applicability of the device. For this reason, soft passive valves are often the preferred option for the integration into microfluidic devices. 

**Figure 6:**
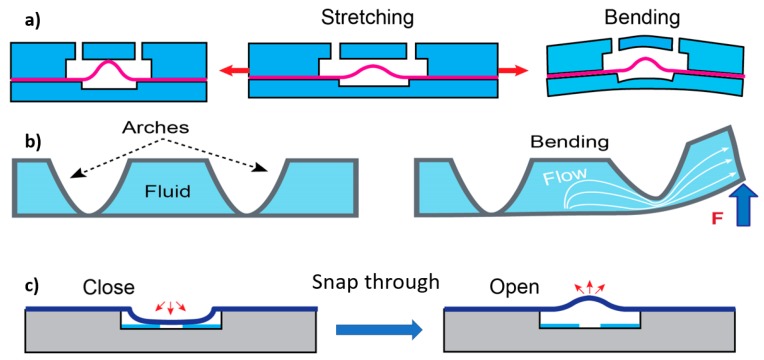
A schematic representation of all flexible microvalves with integrated arches. (**a**) Deflection of the arch under an applied external force such as stretching or bending; (**b**) Integration of two arches in the valve to control over the fluid direction; (**c**) Snap-through concept. The top two concepts differentiate from the bottom one by the source of the applied force.

Holmes et al. fabricated internally valved microfluidic devices by inserting flexible arches in the microchannel [140,141]. The arches were formed by deflecting a thin flexible film that functioned as a valve when they were bent or stretched. As illustrated in Figure 6, under deformation such as bending or stretching, the thin film buckles, enabling the control over the fluid flow. By extending the number of arches, the direction of the flow is controllable using the arch valves. This concept is represented in Figure 6b, in which two arches were embedded in a microchannel. Applying a mechanical stress to one end of the device results in a pressure gradient, forcing the fluid to move from a high-pressure region to a low-pressure region.

Gomez et al. used embedded flexible arches in a channel to achieve passive control in the microfluidic device via elastic snap-through [142]. Snap-through is an instability, where a rapid transition from one condition to another occurs [143,144,145]. The flow of the fluid in a flexible microchannel has a corresponding pressure gradient. The pressure gradient results in snap-through of the embedded arches and allows for passive control of the viscous flow. Figure 6c shows a schematic representation of the embedded arch fabricated from a flexible film, demonstrating how the arch snaps through the two positions in the microchannel [142]. Utilising passive control of the flow by employing flexible materials and exerting snap-through has multiple advantages that can be integrated into a microfluidic system. In this system, the force applied on the arch originates from the fluid flow. Thus, at a specific pressure or flow rate of the fluid, the arch will snap, and this depends on the elasticity of the arch. As such, by manipulating the elasticity of the embedded arch, a microvalve that will open and close at a specific flow rate could be achieved.

### 3.4. Effect of Flexibility on Pumping

Sample delivery is the key task in most lab-on-a-chip systems. A micropump is an indispensable component for generating a smooth rapid flow of the sample fluid. Conventional micropumps are categorised into two groups: Mechanical and non-mechanical pumps. Mechanical micropumps utilise check valves and electrostatic, electroactive, electromagnetic, piezoelectric, and thermal actuators [146]. Non-mechanical micropumps do not have moving parts and are based on electrohydrodynamic, magnetohydrodynamic, electroosmotic, electrowetting and electromechanical concepts [5]. Conventional micropumps are mainly fabricated from silicon and glass with a flexible pump membrane to deliver the driving pressure. 

Conventional micropumps have various drawbacks. First, the fabrication process is complicated, and thus time consuming and expensive. Second, pumping with an external system causes dead volumes in the tubing and is associated with wastage of samples and reagents. Third, the relatively large size of the whole system makes them not economical to be fabricated on a silicon wafer. Furthermore, another important issue with conventional silicon-based micropumps is that they are made of at least two different materials, a rigid chamber and an elastic diaphragm. The two materials need to be bonded well and have the risk of leaking under high pressures. Considering the limitations of conventional micropumps, silicon-based rigid micropumps have been replaced by micropumps made of flexible materials.

Flexible materials are promising candidates for the fabrication of micropumps. For several reasons, flexible micropumps are favoured for the development of microfluidic systems. Elastomeric materials lead to simpler miniaturisation as they do not need any complex fabrication processes [80]. Moreover, soft materials with a low Young’s modulus can deform under a relatively low applied pressure, while conventional micropumps need a powerful external actuating system. Micropumps made entirely of one flexible material are not prone to leaking since the bonded layers are made of the same material. Furthermore, because they are soft and flexible, these micropumps can be implanted inside the human body for applications such as in-vitro drug delivery. Flexible micropumps can reduce sample wastage. As such, flexible polymeric materials find their way into the fabrication and integration of state-of-the-art micropumps [147,148].

Polymers such as poly(methyl methacrylate) (PMMA), poly(carbonate) (PC), poly (amide) (PA), polyimide (PI), poly(vinylidene fluoride) (PVDF), polypyrole, and PDMS have been used for the fabrication of flexible micropumps [103,149,150,151]. These polymers allow making relatively large devices, yet with micrometre resolution. Komatsuzaki et al. [103] reported the fabrication of micropumps entirely out of PI using hot embossing. These PI micropumps are incorporated in microchannels for injecting drugs into human tissues. Flexibility allows the micropump to fit well inside the tissue. Guevara-Pantoja et al. fabricated all-PMMA microfluidic pumps that are thin enough to be flexible [150]. These micropumps can withstand high flow rates and pressures and do not leak as they are made entirely of a single material and well bonded. Bengtsson et al. [149] fabricated soft electroosmotic pumps made of porous polycarbonate coated with PEDOT which is an electrochemically active polymer. The flexible micropump could be incorporated inside a garment for controlling water transport. The advantages associated with micropumps fabricated from polymeric materials are low cost, ideal for mass production, low material consumption, better compatibility with bio/chemical fluids, and ease of fabrication [150].

Peristaltic pumping is another concept that has benefited from flexibility. Flexible materials are most suited for the peristaltic pumping concept [152,153,154]. A peristaltic pump utilises either the flexible channel wall or synchronised pumping chambers to deliver fluid flow. Common actuators for peristaltic pumps include shape memory alloy, electromagnetic, piezoelectric, electrostatic, and thermopneumatic actuators [155]. Ma et al. reported a piezoelectric peristaltic micropump with a low dead volume in the microchannel [152]. 

Another new type of flexible micropump emerged recently is called bio-actuated micropump. The working mechanism of these pumps is based on the contractile force of cardiac muscle cells which provide actuation. Shutko et al. [156] reported a biologically actuated thin flexible micropump made of PDMS. The pump delivers fluid flow by taking advantage of the peristaltic movements of the flexible channels.

**Figure 7:**
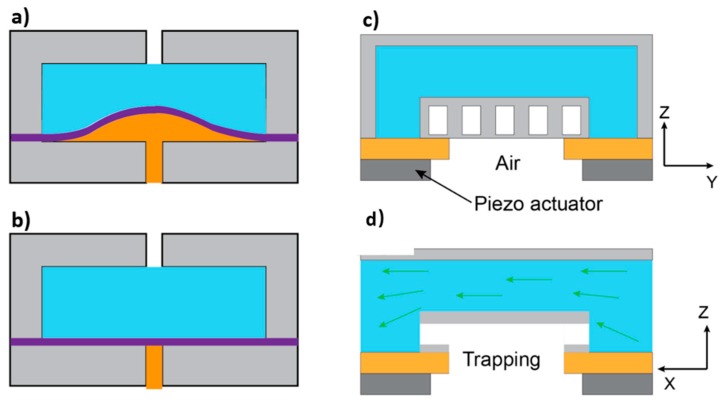
The concept of flexible hydraulic reservoir (FHR): (**a**) The sample is pumped by the pressure applied to the actuating fluid (blue); (**b**) The sample is delivered without any dead volume; (**c**) Acoustofluidic flexible micropump. The piezo ring that provides the excitation for the flexible layers; (**d**) Cell trapping with the micropump.

In addition to flexible peristaltic micropumps, other types of stand-alone flexible micropumps have been reported. One example is the flexible hydraulic reservoir (FHR) [157]. Figure 7a,b show that the pump simply comprises of a flexible membrane situated in a fluid chamber. One part of the chamber can be filled with any actuating liquid while the other part is filled with the sample to be pumped. The actuating liquid pushes the membrane under the pressure, thus the sample is delivered into the microchannel with zero dead-volume. FHR could be used for high cost or rare liquids transfusion.

Very recently, a new type of acoustofluidic flexible micropump has been fabricated and reported by Lin et al. [158]. Figure 7c,d illustrate the schematic of this micropump. The top layer consists of the microchannels, the inlet and the outlet, which is made from flexible PDMS. The bottom layer is a piezo actuator. This non-mechanical micropump transforms acoustic energy provided by a piezo ring into movement of the flexible layers. Due to the low pressure, a strong bond between the two layers is not required. As such, the micropump has been designed for the stick-and-play mode, where the top layer could be removed and reused several times. This flexible micropump can be integrated into flexible microfluidic devices used in lab-on-a-chip applications for single-cell trapping.

### 3.5. Effect of Flexibility on Separation

In areas such as translational medicine, diagnostics, chemical analysis, food, and environment, there exists a priority in functionality to separate, sort, and purify microparticles in a fluid flow. For example, cancer cells or dead cells need to be separated from healthy cells [13]. In chemical industry, unwanted impurities need to be extracted from the product. Microfluidics offer solutions for continuous separation and sorting of microparticles [20]. Microfluidic separation techniques are categorised into either active or passive types. Active techniques utilise external energy and are based on magnetophoresis, optical trapping, dielectrophoresis, and acoustophoresis to name a few [12]. Passive techniques such as deterministic lateral displacement, pinched flow fractionation, inertial microfluidic, hydrophoresis, viscoelastic microfluidics, and micro vortex trapping make use of the geometry and the structure of the microchannels, particle interactions, flow field, and the equilibrium between the forces and the interactions in the channels [20,159,160]. Combining active and passive methods, hybrid systems have been developed to take advantage of both approaches to improve the separation efficiency [161,162,163].

Among the above-mentioned methods for microparticle separation, inertial microfluidics has gained much attention and has benefited from the concept of flexible microfluidics. In inertial microfluidics, particles are separated based on the equilibrium of the inertial lift forces and Dean drag forces [161,164,165,166,167,168]. Dean flow, normally observed in a curved channel, is a rotational flow perpendicular to the main flow which applies an extra drag force on the particles, leading to sorting of the particles based on their sizes. In addition to the effect of the different forces, flow rate also plays an important role in inertial microfluidic separation. Since flexibility of the microchannel significantly influences the flow rate as discussed earlier in Section 3.1., we hypothesize that flexibility can affect the separation and sorting of the particles and presents a research gap to be studied. Employing inertial microfluidics in 2D and 3D curved or tubular microchannels for separation and sorting microparticles have been conducted using flexible microfluidics. Spiral microchannels employing flexible polymer tubing have been utilised for separation purposes. Soft tubing provides a high degree of freedom for channels to rearrange themselves. Researchers have used these reconfigurable deformable tubes to improve separation and sorting of the microparticles. 

**Figure 8:**
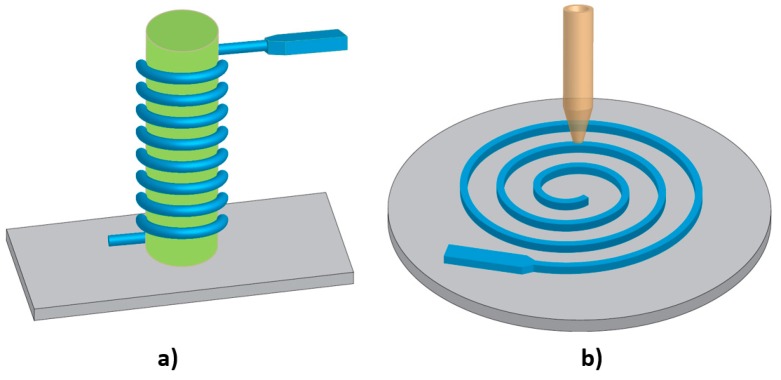
Schematic illustration of soft microtubes with two configurations: (**a**) 3D, the tubular microchannels are coiled around a rod; and (**b**) 2D, the tubular microchannels are placed in a plane.

Two techniques have been utilised in fabricating 3D spiral microchannels. The first technique is fabricating elastic microtubes and coiling them in a three-dimensional (3D) configuration as depicted in Figure 8a. These soft microtubes can also be used in two-dimensional (2D) systems, if they are placed in a plane as shown in Figure 8b. Xi et al. [169] developed flexible microtubes by pulling a heated metal wire through a pool of PDMS precursor and crosslinker. The PDMS was cured around the wire and was later separated from the metal wire through sonication. Hahn et al. [170] used flexible polymer tubes coiled around a rod to separate particles of different sizes. The fabricated microtubes are flexible and stretchable. The flexible platform is simple, easy to fabricate, and efficient in separation. Another advantage of these microtubes is the possibility of mimicking blood vessels and studying the flow of blood and separation of blood cells in these microtubes, as they have a comparable size and geometry to human blood vessels. Additionally, elastic microtubes could be utilised in fabrication of wearable microfluidic sensors as they can be integrated with liquid metals [171]. These tubes can also be used as force sensors due to the high sensitivity of the flexible structure.

**Figure 9:**
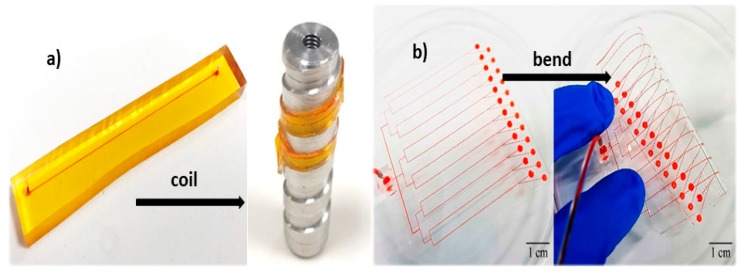
Three-dimensional flexible microchannels: (**a**) Coiling a planar 2D microchannel around a rod. Adapted with permission from Asghari et al. [172]; (**b**) Bending a flexible planar microfluidic channel. Adapted with permission from Jung et al. [90].

The second technique for fabricating 3D flexible spiral microchannels is making planar 2D microchannels and then coiling them around a rod into 3D structures (Figure 9a) [172]. Also, Jung et al. [90] have proposed an interesting method in producing 3D microchannels by simply bending a flexible planer microfluidic channel (Figure 9b). This method further provides the possibility of adjusting the channel curvature and therefore tuning the Dean flow inside the flexible microchannels. Thus, this method allows for controlled inertial separation. 

## 4. Recent Applications of Flexible Microfluidics

The emerging field of flexible microfluidics presents a paradigm shift in traditional application domains such as drug delivery, cell analysis, organ-on-a-chip, lab-on-a-chip, wearables and biosensors, neural probes, biomolecule separation, and microanalysis. In addition to the flow control applications including micropumps, micromixers, microvalves, and micro separators previously discussed in earlier sections, flexible microfluidics offer novel applications in flexible and wearable electronics as well as biology. Advances in these new applications will be discussed in the following subsections.

### 4.1. Flexible and Wearable Electronics

#### 4.1.1. Mechanical Sensors

Recently, several microfluidic-based flexible wearable sensors that detect pressure, tactile, and shear forces for use in biomedical as well as robotic applications [81,173,174,175] have emerged. These sensors are based on channels filled with a liquid metal alloy usually eutectic gallium-indium (EGaIn) that serves as the sensing component. Flexible wearable sensors require specific features such as a defined thickness, a relatively low Youngs modulus, compatibility with the skin, stretchability, and conformality on the skin [174]. Novel approaches for fabricating flexible wearable electronics based on microfluidics will now be further explored.

**Figure 10:**
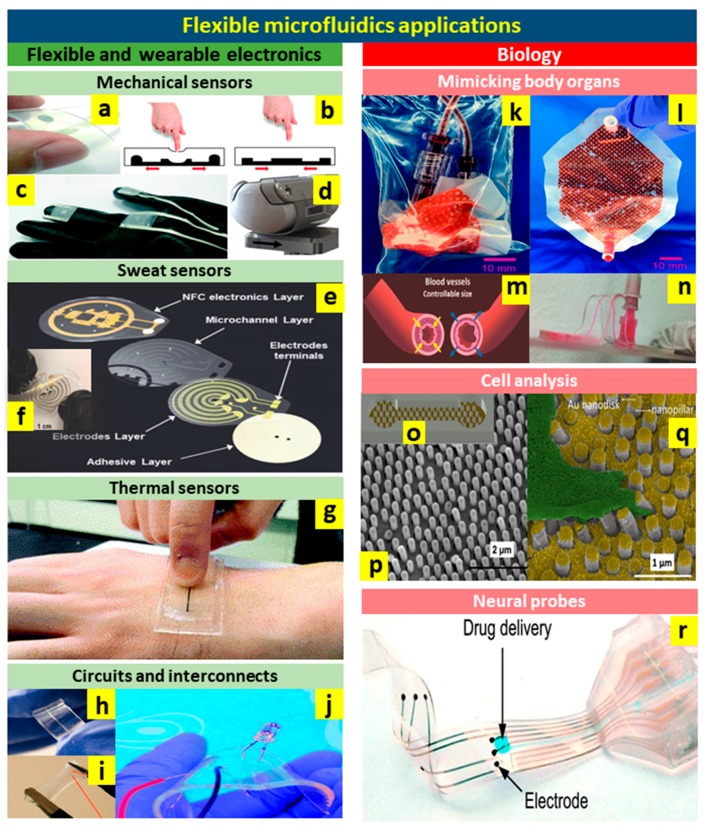
Applications of flexible microfluidics. The left side illustrates applications in flexible wearable electronics. The right side illustrates the applications in biological devices. (**a**–**c**) Pressure tactile sensors. Adapted with permission from Yeo, et al. [27]. (**d**) Flexible microfluidic shear force sensor wrapped around an artificial fingertip for shear measuring. Adapted with permission from Yin, et al. [81]. (**e**,**f**) Flexible microfluidic sweat sensor fabricated from several layers. Adapted with permission from Kim, et al. [177]. (**g**) Microfluidic wearable temperature sensor placed on the skin. Adapted with permission from Yoon, et al. [180]. (**h**,**i**) Microfluidic-based flexible stretchable circuits. Adapted with permission from Alfadhel, et al. [176]. (**j**) Flexible conductive microfluidic circuit. Adapted with permission from Sun et al. [181]. (**k**,**l**) Artificial placenta-type blood oxygenator based on flexible microfluidics. Adapted with permission from Dabaghi et al. [48]. (**m**,**n**) Elastomeric multidimensional microchannel inspired by blood vessels. Adapted with permission from Wu, et al. [185]. (**o**–**q**) Flexible PDMS microchannels integrated with polymeric nanopillars covered with gold nanodisks and fibroblasts spreading on them. Adapted with permission from Solis-Tinoco, et al. [46]. (**r**) Represents a microfluidic-based flexible neural probe. Adapted with permission from Minev et al. [189].

A flexible microfluidic device with EGaIn-filled microchannels was produced by Yeo et al. [27] as a pressure tactile sensor, Figure 10a. Applying force on the pressure sensing regions of the device, Figure 10b resulted in the deformation of the microfluidic channels underneath, leading to a change in the resistance of the device. To demonstrate the capability of object grasping, the pressure sensors were embedded in a glove, Figure 10c. The resulting data showed consistency and reproducibility in the response of the pressure sensor as well as a good sensitivity of 0.05 kPa^−1^. The reliable and consistent performance of the sensors under various distortions further confirmed their robustness and functionality. Beside the pressure, shear force is another important parameter that needs to be measured for achieving a precise 3D-tactile sensing. 

Inspired by the layered structure of the skin, Yin et al. reported a shear force sensing skin based on flexible PDMS microfluidics [81]. This resistive sensor was made of two thin layers of PDMS bonded through plasma treatment. The channels were then filled with EGaIn, functioning as a strain sensor. As a demonstration, the sensor was wrapped around an artificial fingertip as depicted in Figure 10d. By applying a shear force to the skin sensor, the compression and the tension induced in the skin was measured via strain gauges. The flexible sensor was conformal and could be mounted on skin. The shear sensor exhibited an accuracy of 0.08 N, which is very near to the acceptable sensitivities used for robotic purposes. 

In addition to soft-lithography technique used for the construction of microfluidic sensing devices, recent advanced inkjet printing technologies have also been employed to manufacture flexible microfluidic devices for sensing purposes [176]. In a recent study by Alfadhel et al. [176], a microfluidic resistor exhibiting high stretchability and foldability with controllable shapes and dimensions was fabricated. In this work, polyethylene glycol (PEG) used as a sacrificial ink was simply deposited on a flexible substrate. Next, the top layer was drop cast on the template, cured and cooled. Later, the template was washed away at its phase-change temperature. The fabricated device with the potential for sensor integration was later used to produce flexible resistors by filling the microchannels with EGaIn. Deformations applied to the device lead to a change in the shape of the microchannels, resulting in a resistance change. These resistors could be employed for strain, tactile, and pressure sensing.

#### 4.1.2. Sweat Sensors

Employing flexible microfluidics in wearable electronics also overcomes the limitations associated with conventional sweat sensors. Recently, Kim et al. [177] reported a battery-free wearable sweat sensor utilising flexible microfluidics with wireless readout using radio frequency (RF) communication, Figure 10e,f. The device was integrated with electrodes along the microchannels to measure the change in resistance associated with changes in the constituents of the sweat. The electrode was fabricated using a PI spin-coated substrate with Cu and Au deposited on it and then patterned into favourable shapes. The microchannels were fabricated by soft lithography of PDMS. The near field communication (NFC) layer was constructed from PDMS with integrated magnets. The device enabled continuous measurement of time-dependant sweat markers.

#### 4.1.3. Temperature Sensors

Highly accurate wearable temperature sensors have shown great potential for medical diagnosis and advanced health care monitoring applications. Examples of flexible microfluidic-based temperature sensing devices include liquid-state heterojunction sensors [178], and micro-thermocouples [179] using liquid metal alloys [180]. These microsensors overcome the impediments affiliated with solid-metal thin-film sensors such as complex fabrication processes, brittleness, and being vulnerable to high temperatures. For instance, Ota et al. [178] reported highly flexible liquid-state heterojunction temperature sensors based on all PDMS microfluidics that can sustain various types of deformations without any functionality loss. Various types of ionic liquids with unique ion transfer assets have been employed in these sensors to perceive different incentives. Due to the utilisation of different types of ion liquids, the sensors exhibit high-temperature sensitivities. Figure 10g illustrates a microfluidic wearable temperature sensor [180].

#### 4.1.4. Flexible Circuits and Interconnects

In addition to sensing elements, circuitry and interconnects with excellent bendability and stretchability play an important role to construct a fully wearable device. Figure 10h–j represent some microfluidic-based flexible stretchable circuits and interconnects [176,181]. The use of fluidic interconnects enables extremely large deformation and stress, thereby eliminates the crack issues that usually occur in solid-metal-based components. Sun et al. [181] reported the fabrication of flexible conductive microfluidic circuits with potential applications in wearable sensors and implantable biomedical devices, Figure 10j. PDMS was first treated using poly(vinyl alcohol)/glycerol (PVA/Gly), followed by the deposition of silver nanowires (AgNWs) on the microfluidic channels. Since AgNWs [182,183] are highly electrically conductive at low concentrations, electrodes with great transparency could be achieved using AgNWs. This can be considered a significant advantage of using AgNWs since conventional conductive materials used for flexible devices such as carbon nanotubes [184] exhibit relatively poor transparency. Experimental results also demonstrated the long-term stability of AgNW-based flexible microfluidic circuits over various deformations.

### 4.2. Biology

Flexible microfluidics provides unprecedented functionality which can revolutionise biomedical technologies. The flexibility of microfluidics overcomes the mechanical mismatch between the conventional rigid electronics and bio-tissue, making this platform highly relevant for implantable applications. The deformability of microfluidics as well as their permeability also well represent the general conditions of living organisms. 

Flexible microfluidics has found their applications in several biological areas. For instance, Dabaghi et al. [48] reported an artificial placenta-type blood oxygenator based on flexible microfluidics, Figure 10k,l. The device allows for enhanced gas exchange to assist premature neonates who suffer from respiratory distress syndrome. This device consists of networks of crossing microchannels made of PDMS with high gas permeability reinforced with porous PTFE membranes. Flexible microfluidics allows for the implementation of a robust blood oxygenator that could be folded several times without any breaks or leakage. The device maintained its functionality under several cycles of bending and distortions. Wu et al. [185] reported an elastomeric multidimensional microchannel inspired by blood vessels, in which 3 M VHB elastomer—a double-sided very high bond tape—and PDMS were used to make the stretchable microchannels (Figure 10m,n). The team investigated the elasticity and mechanical properties of the microchannels under different conditions such as stretching and relaxing. The microchannels exhibited great mechanical properties in very high stretch rates, showing that they have great potential for strain sensors and flow regulators. Flexible PDMS microparticles have been designed for biomicrofluidics applications [186]. Flexible transparent PDMS microparticles were produced to mimic red blood cells to be later evaluated based on their flow behaviour [187]. Rheological measurements of the solution with these microparticles revealed that they show the same shear-thinning behaviour of human blood. The deformation of these microparticles were used to study the diseased red blood cells.

Flexible microfluidics has also been used for cell analysis [188]. Solis-Tinoco et al. [46] designed a flexible microfluidic device to study cell adhesion. The PDMS microchannels were integrated with polymeric nanopillars covered with gold nanodisks, Figure 10o,p. The pillars were fabricated from both soft and rigid polymers to resemble different types of tissues. Mechanical properties of the optical biosensor such as Young’s modulus and spring constant of the pillars were adjusted to investigate cell contractile forces. Adhesion and spreading of fibroblasts, cells responsible for making the extracellular matrix and collagen, could be observed and studied using this flexible microfluidic device, Figure 10q.

Soft neural probes are another application of flexible microfluidics in biology. The current hard probes are incompatible with neural tissues due to their mechanical mismatch with soft bio-tissues. The long-term use of hard probes in neural tissues is associated with many issues such as neuroinflammation. Flexible microfluidic platforms fabricated using PI, parylene, PDMS, and SU-8 have been utilized in the production of soft neural probes to overcome the drawbacks of the hard probes [86,87,98,189,190]. These soft devices exhibit excellent conformation and adaptability to biological tissues. Recent advances in flexible microfluidic neural probes have been reviewed by Sim et al. [191]. Minev et al. [189] have integrated flexible microfluidics into soft neural probes, Figure 10r. These soft probes maintain various mechanical deformations, electrical surges, and chemical injections as well as meeting the required attributes of spinal and brain tissues.

## 5. Future Perspectives

Flexible microfluidics’ characteristics show promise in properties that adapt to a variety of functions and applications. In order to fully reveal its potential, a great effort still needs to be made. As already stated, siloxane elastomers are the most used materials for flexible microfluidic fabrication. However, to date, only PDMS has been extensively studied. Mechanical, chemical, and biological properties of other siloxane elastomers such as Ecoflex that could have better attributes regarding flexibility and stretchability, have not been well understood yet. The characteristics of these elastomers are largely affected by their composite such as crosslinking content as well as their dimensions such as thickness. Therefore, comprehensive investigations are required to elucidate the properties and potential of these flexible materials. 

In flexible electronics, a device needs to be highly conformal to the skin in order to be wearable. Many of the materials so far used for producing microfluidic wearable sensors are not sufficiently conformal due to their intrinsic mechanical properties. If wearable microfluidic devices are made of materials with high conformability without wrinkling, folding or moving on the skin, they can sense the smallest movements and vibrations. Recently, hydrogels have been used for microfluidic applications since they exhibit a high degree of stretchability [192,193]. Hydrogels can conform to the skin very well. As such, hydrogels are expected to be one of the prospective materials for the future stretchable wearable microfluidics.

Another research gap relevant to note is regarding the hybrid materials, particularly for electrically conductive flexible and stretchable microfluidic sensors. Recently, a new technique for in-situ polymerization of PEDOT:PSS inside the microchannels has been proposed [194]. This technique allows for the fabrication of stretchable microfluidic tubes with conductive polymers coated in the inner wall of the microchannels. 

Wearable electronics have rapidly evolved and are expected to become one of the biggest industries. The tremendous number of devices could lead to disposal issues. Therefore, flexible microfluidic devices with the ease of decomposition after use could greatly benefit this purpose. To date, most flexible and stretchable microfluidic devices are made of non-biodegradable materials. The only biodegradable material that has been used so far is paper. However, paper material lacks stretchability and gas and water permeability, which limits its application in microfluidics. Biodegradable polymers such as chitosan and poly (lactic acid) (PLA) could be promising candidates for the purpose of disposable flexible microfluidics. In order to validate their suitability for flexible microfluidics, their mechanical properties, fabrication and sealing methods, biocompatibility, and compatibility with other materials need to be systematically studied.

## 6. Conclusions

Flexible microfluidics is a multifunctional and multidisciplinary field that finds its way into many research areas including biology, electronics, chemistry, and medicine. Having a clear idea about the fundamentals and recent advances in this area allows researchers to have a better perspective. The present review paper discusses the fundamentals, materials, applications, and future of flexible microfluidics. In the first part, following a brief review of flexibility and stretchability, materials for flexible microfluidic devices were introduced and discussed in detail. Also, advantages, disadvantages, and the opportunities that each material may bring were raised. In the second section, discussion around the effect of flexibility on different microfluidic parameters and functions such as flow rate, pressure drop, valving, pumping, mixing, and separation were explored. As the fluid passes through the flexible microdevice, the channels experience a deformation caused by the fluid pressure. The deformations coupled with fluid-wall interactions lead to unique phenomena that affect the microfluidic functions. The pressure-drop of the fluid passing through a flexible microchannel is less than that of a rigid channel. As such, flexible microchannels can stand higher flow rates without leaking. Mixing can be improved in flexible microchannels due to the instabilities induced by fluid-wall interactions. Novel approaches toward the construction of all flexible microvalves and micropumps were presented and discussed. Regarding separation, a detailed summary of curved and spiral microchannels fabricated via flexible microfluidics for separation purposes was explained. The third section discussed the recent applications of flexible microfluidics in wearable electronics and biology. Flexible microfluidics have been utilised for the fabrication of wearable sensors by employing a flexible material as well as a liquid metal alloy inside the microchannels. We summarised several novel microfluidic wearable devices for pressure, tactile, shear, strain, temperature, and sweat sensing. The last section of the review discussed the promising future directions regarding flexible microfluidics. Flexible microfluidics is still at its infancy and the shape of the field is still not clear. Further scientific efforts need to be made to take full advantage of flexibility and exploit its application for microfluidic functions, wearable sensing, and even implantable therapy.

## Figures and Tables

**Table 1:** List of common materials for flexible microfluidics.

Materials	Tg ^1^ (°C)	Young’s Modulus	Advantages	Disadvantages	Fabrication Techniques	References
PDMS	−125	<1000 kPa	low Tg, low shear and Youngs modulus, high optical transparency, durability, gas permeability	hydrophobic nature, incompatibility with solvents	soft lithography, plasma-enhanced bonding	[63,69,70,73,74]
Ecoflex	NA	40 kPa	low Young’s modulus, highly flexible, high tear strength, and large elongation	non-transparent, high viscosity, incompatible with plasma bonding	soft lithography	[27,77,78,114,115]
Parylene	<90	2.7–4 GPa	biocompatibility, low water absorption, transparent, solvent resistance, surface conformality	costly, complicated fabrication, hard to handle, low adhesion to substrates	vapor deposition bonding	[84,85,86,87,88,89,90,116,117]
PI	300–400	2.5 GPa	biocompatibility, high thermal stability, good sealing properties, chemical inertness	opaque, moisture absorption	lamination, sacrificial layer techniques, wet/dry etching, hot embossing	[93,94,95,96,97,98,103,104,116]
OSTE	25–70	0.25–2 GPa	scalable commercial production possibility, low polymerization shrinkage stress, direct lamination and bonding	very high OS ratios can lead to residual monomers that may affect cells and proteins	soft lithography	[105,106,108,118]
PET	69–78	2–2.7 GPa	good gas and moisture barrier properties, chemically inert, recyclable	poor chemical resistance, needs surface treatment for bonding due to the low plasma bonding strength	moulding by hot embossing, thermal bonding	[27,91,92,119]

^1^ Glass transition temperature.
